# Pharmacotherapy for non-infectious uveitis: spotlight on phase III clinical trials of locally injected or implanted therapeutics and systemic immunomodulatory drugs

**DOI:** 10.1186/s12348-025-00502-9

**Published:** 2025-06-05

**Authors:** Melissa K. Shields, Lisia Barros Ferreira, Syed B. Ali, Liana Dedina, Lyndell L. Lim, Eric B. Suhler, Justine R. Smith

**Affiliations:** 1 College of Medicine and Public Health, and Flinders Health and Medical Research Institute, Flinders University, Adelaide, South Australia Australia; 2 Centro de Medicina Humanizado, Curitiba, Paraná Brazil; 3 Flinders Medical Centre, Southern Adelaide Local Health Network, Adelaide, South Australia Australia; 4 Centre for Eye Research Australia, Melbourne, Victoria Australia; 5 Casey Eye Institute, Oregon Health & Science University, and Portland Veterans’ Affairs Health Care System, Portland, OR USA

**Keywords:** Non-infectious uveitis, Phase III clinical trial, Drug, Local, Systemic, Corticosteroid, Immunomodulatory

## Abstract

**Background:**

Non-infectious uveitis is an uncommon eye condition, but an important cause of substantial vision impairment and blindness around the world. An individualized treatment approach may include corticosteroids, conventional immunomodulatory medications, and biologic immunomodulatory agents, delivered locally to the eye or by systemic routes.

**Body:**

In this narrative review, we describe pivotal phase III clinical trials that have provided the information guiding clinical practice in non-infectious uveitis today, focusing on studies of injected or surgically positioned local therapeutics, systemic immunomodulatory drugs, and the combination of both. We report on design, outcome measures, and key effectiveness and safety results of these trials. We also describe selected phase III clinical trials that are presently in progress, with results expected within the coming approximately 3 years. Finally, we summarize the state of the field and speculate on fruitful areas of research and development for new phase III clinical trials of drugs to treat non-infectious uveitis.

**Conclusion:**

The locally delivered therapeutics for non-infectious uveitis that have been studied in phase III clinical trials are largely corticosteroid-based. Trials have shown these locally placed corticosteroids to be effective, with complications that include elevated intraocular pressure and cataract. A minority of systemic immunomodulatory drugs used to treat non-infectious uveitis have been studied in phase III clinical trials. Key information obtained from those trials includes the following: similar effectiveness of methotrexate and mycophenolate mofetil for uveitis involving the posterior eye; usefulness of interferon-beta for intermediate uveitis; and potent anti-inflammatory activity of adalimumab in recalcitrant uveitis. Phase III clinical trials of immunomodulatory drugs with novel targets or routes of delivery can be anticipated in the foreseeable future. Interest in defining treatments for specific subsets of non-infectious uveitis through phase III clinical trials is also likely. For productive cross-referencing of clinical trial results, we recommend the use of core outcome measures.

## Background

Non-infectious uveitis is a large group of eye diseases, caused by diverse pathological mechanisms but all characterized by the presence of intraocular inflammation [1]. The inflammation affects different compartments within the eye, which is indicated by ‘Standardization of Uveitis Nomenclature’ terminology; the ‘Anatomic Classification of Uveitis’ includes anterior uveitis (based in the anterior chamber), intermediate uveitis (based in the vitreous), posterior uveitis (based in the retina and/or choroid), and panuveitis (involving all compartments) [2]. Different types of uveitis may be undifferentiated – commonly referred to as idiopathic – or they may represent a recognized ocular syndrome or be associated with a systemic inflammatory disease [3]. Individually these different conditions are uncommon or rare, but as a group they are an important cause of blindness and impaired vision across low, middle, and high income countries [4]. Vision loss in patients with uveitis may be caused by the inflammation itself, or associated complications such as cataract, glaucoma, and macular edema [5].

The treatment of non-infectious uveitis is typically individualized, and often involves a team approach [6]. Other medical specialists, including immunologists, rheumatologists, and general internists or pediatricians, work together with ophthalmologists and their patients to plan the optimal treatment. Drugs include corticosteroids, conventional immunomodulatory medications, and biologic immunomodulatory agents [7]. Some drugs may be delivered locally, as eye drops, periocular or suprachoroidal injections, and intravitreal injections or implants [8]. Other drugs are delivered systemically, by oral, subcutaneous and intravenous routes. Considerations in treatment-planning include the specific type of uveitis, its laterality and location of the inflammation within the eye (which is relevant for the mode of drug delivery), the age of the patient, and co-morbidities and life-style factors. The current or future threat to vision is another important factor to be considered. Typically when the vision is threatened, oral corticosteroids are initiated early and briefly to achieve prompt control of the inflammation, with other therapies substituted for long-term management [6]. In some situations, corticosteroid eye drops, or injected or surgically positioned local corticosteroid therapeutics are the first-line treatment.

Over the past approximately 20 years, the international community of uveitis-specialized ophthalmologists have joined forces, in some instances in collaboration with the pharmaceutical industry, to evaluate local therapeutics and systemic drugs for the treatment of non-infectious uveitis. In this narrative review, we describe selected published pivotal phase III clinical trials that have provided the information that guides clinical teams and their patients today. Included articles were identified from literature searches conducted between February 13, 2024 and December 16, 2024, using the National Library of Medicine (NLM) PubMed database and multiple terms used in various combinations: *uveitis*, *non-infectious uveitis*, *autoimmune uveitis*, *treatment*, *trial*, *clinical trial*, *phase 3*, *phase III*, and *randomized*. To identify new clinical trials, the NLM ClinicalTrials.gov database was interrogated with the search term, *uveitis*, while applying the *recruiting and not yet recruiting* filter. Additional focused searches were conducted using names of specific therapies, and manual reference mining of articles was also used to identify relevant publications. Finally, the senior co-authors (LLL, EBS and JRS) provided insights for the selections from their first-hand involvement in clinical trials in the field.

To support the reader experience, we have grouped the selected clinical trials based on whether they were investigations of local therapeutics, systemic treatments, or a combination of both. With a spotlight on sight-threatening posterior eye involvement, we do not address clinical trials of eye drops for non-infectious uveitis. Our review ends with a section that describes some clinical trials that are presently in progress, with results anticipated in the next approximately 3 years. Uveitis can be caused by infections or rarely may represent a malignancy masquerading as inflammation [9, 10]. These forms of uveitis are not considered in our review. However, we wish to highlight that infection and malignancy are considerations at the initial uveitis assessment, and must be ruled out before the initiation of immunomodulatory drug therapy.

## Phase III clinical trials of therapeutics delivered locally to the eye

Corticosteroids delivered to the posterior segment of the eye effectively control inflammation in non-infectious intermediate, posterior, and pan- uveitis. These therapeutics include intravitreal fluocinolone acetonide and dexamethasone implants, and the suprachoroidal triamcinolone acetonide injection. Other locally delivered drugs, such as intravitreal injection of methotrexate, sirolimus, and ranibizumab, have more limited evidence of effectiveness, but may avoid common side effects associated with the corticosteroid therapeutics. This section reviews the evidence from phase III clinical trials for injected or surgically positioned local therapeutics in the management of non-infectious uveitis. Table 1 summarizes the design features of these studies, as well as the main effectiveness and safety results.

**Table 1:** Summary of phase III clinical trials of therapeutics delivered locally to the eye for non-infectious uveitis

Identification number (name), year [Ref]	Design (duration) and location	Study population	Treatment groups	Main effectiveness results	Main safety results
**Intravitreal fluocinolone acetonide implant**					
NCT00407082, 2008 [11]	Prospective, multicenter, double-masked, historically controlled RCT (36 months) United States, Singapore	278 patients ≥ 6 years with recurrent unilateral or bilateral PU ≥ 1 year, and ≥ 2 recurrences or requiring systemic corticosteroids for ≥ 3 months or ≥ 2 sub-Tenon’s corticosteroid injections within past 6 months	**Active**: 0.59 mg FAi (*n* = 110) **Active**: 2.1 mg FAi (*n* = 168) [Original implant (*n* = 70), Redesigned implant (*n* = 98)]	**Cumulative uveitis recurrence rate (1-year pre-implantation**,** 1-**,** 2-**,** 3-year) (%)**: 0.59 mg FAi: 62, 4, 10, 20 (*p* < 0.01); 2.1 mg FAi (original): 71, 7, 27, 57 (*p* < 0.01 except *p* = 0.06 at 3 years); 2.1 mg FAi (original): 49, 6, 9, 29 (*p* < 0.01) **Mean time to first recurrence (months ± SD)**: 0.59 mg FAi: 33.5 ± 0.6; 2.1 mg FAi (original): 29.3 ± 1.1; 2.1 mg FAi (redesigned): 32.6 ± 0.8; Non-implanted eyes: 19.9–20.1 ± 1.2–1.5 **≥ 15-letter increase in BCVA from baseline at 36 months (%)**: 0.59 mg FAi: 23 (*p* < 0.01); 2.1 mg FAi: 18 (*p* < 0.01) **Eyes experiencing reduction in area of ME at 12 months**,** 36 months (%)**: 0.59 mgFAi: 86, 73 (*p* < 0.01); 2.1 mg FAi: 70, 45 (*p* < 0.01) **Use of systemic adjunctive medications (1-year pre-implantation**,** 1-**,** 2-**,** 3-year) (%)**: 0.59 mg FAi: 44, 7, 12, 8 (*p* < 0.01); 2.1 mg FAi: 57, 14, 11, 13 (*p* < 0.01) **Use of periocular corticosteroid injections (1-year pre-implantation**,** 1-**,** 2-**,** 3-year) (%)**: 0.59 mg FAi: 69, 4, 7, 13 (*p* < 0.01); 2.1 mg FAi (combined): 67, 2, 11, 32 (*p* < 0.01)	**Cumulative IOP elevation from baseline ≥ 10 mmHg (%)**: 0.59 mg FAi: 67; 2.1 mg FAi: 79 **Glaucoma surgery (%)**: Implanted eyes (combined 0.59 mg and 2.1 mg FAi groups): 40; Non-implanted eyes: 2 (*p* < 0.01) **Cataract surgery (%)**: Implanted eyes (combined 0.59 mg and 2.1 mg FAi groups): 93; Non-implanted eyes: 20 (*p* < 0.01) **Hypotony (< 6 mmHg) (%)**: 0.59 mg FAi: 34; 2.1 mg FAi: 46; Non-implanted eye: 15 (*p* < 0.01)
NCT0456482, 2015 [12]	Prospective, multicenter, double-masked, dose-controlled RCT (36 months) India, Canada, Australia, United States, Hong Kong, Philippines	239 patients ≥ 6 years with recurrent, unilateral or bilateral PU ≥ 1 year and ≥ 2 recurrences or requiring systemic corticosteroids for ≥ 3 months or ≥ 2 sub-Tenon’s corticosteroid injections within past 6 months	**Active**: 0.59 mg FAi (*n* = 117) **Active**: 2.1 mg FAi (*n* = 122)	**Cumulative uveitis recurrence rate (1-year pre-implantation**,** 1-**,** 2-**,** 3-year) (%)**: 0.59 mg FAi: 43.6, 12.8, 13.7, 17.1 (*p* < 0.0001); 2.1 mg FAi: 41, 12.3, 18, 34.4 (*p* < 0.0001 years 1 and 2, *p* = 0.2850 year 3); Non-implanted eyes: 19.8, 49.6, 57.6, 59.7 (*p* < 0.0001) **Mean time to recurrence (months ± SD)**: 0.59 mg FAi: 30.1 ± 1.0; 2.1 mg FAi: 30.5 ± 1.0; Non-implanted eyes: 17.3–18.1 ± 1.4 **≥ 15-letter increase in BCVA from baseline at 36 months (%)**: 0.59 mg FAi: 31.1; 2.1 mg FAi: 23.6; Non-implanted eyes: 9.5 (*p* ≤ 0.0046) **Eyes experiencing reduction in area of ME at 36 months (%)**: 0.59 mg FAi: 76.0 (*p* < 0.0001); 2.1 mg FAi: 62.5 (*p* < 0.0001); Non-implanted eyes: 20.4 (*p* < 0.0001) **Use of systemic adjunctive medications (1-year pre-implantation**,** 1-**,** 3-year) (%)**: 0.59 mg FAi: 63.2, 13.9, 12.0 (*p* < 0.0001); 2.1 mg FAi: 59.0, 9.2, 13.1 (*p* < 0.0001) **Use of periocular corticosteroid injections in study eye (1-year pre-implantation**,** 1-**,** 3-year) (%)**: 0.59 mg FAi: 55.6, 6.8, 9.4 (*p* < 0.0001); 2.1 mg FAi: 62.3, 6.6, 24.6 (*p* < 0.0001)	**Cumulative IOP elevation from baseline ≥ 10 mmHg (%)**: 0.59 mg FAi: 67.8; 2.1 mg FAi: 71.3 **Cataract surgery (%)**: 94.9 (combined 0.59 mg and 2.1 mg FAi groups) **Hypotony (< 6 mmHg) (%)**: 0.59 mg FAi: 40.2; 2.1 mg FAi: 61.5 *(p* = 0.007)
NCT01694186, 2020 [13]	Prospective, multicenter, double-masked, sham-controlled RCT (36 months) United States, United Kingdom, Germany, Hungary, Israel	129 adult patients with IU, PU or PanU in ≥ 1 eye for ≥ 1 year and ≥ 2 recurrences of uveitis requiring systemic corticosteroid, immunosuppressive treatment, or intraocular corticosteroid	**Active**: 0.2 µg/day FAi (*n* = 87) **Control**: Sham injection + standard of care (*n* = 42)	**Proportion of patients with uveitis recurrence in study eye (6-months**,** 1-**,** 3-years) (%)**: FAi: 27.6, 37.9, 65.5; Control: 90.5, 97.6, 97.6 (*p* < 0.001) **Median time to first recurrence (days)**: FAi: 657; Control: 70.5 (*p* < 0.001) **≥ 15-letter increase in BCVA from baseline at 36 months (%)**: FAi: 33.3; Control: 14.7 **Patients receiving ≥ 1 adjunctive systemic medication (%)**: FAi: 34.5; Control: 50.0 **Patients receiving ≥ 1 local corticosteroid injection (%)**: FAi: 19.5; Control: 69.0	**IOP events > 25 mmHg**,** > 30 mmHg (%)**: FAi: 24.1, 16.1; Control: 23.8, 11.9 **Glaucoma surgery (%)**: FAi: 5.7; Control: 11.9 **Cataract surgery (%)**: FAi: 73.8; Control: 23.8 **Hypotony (< 6 mmHg) (%)**: FAi: 10.3; Control: 11.9
NCT02746991, 2023 [14]	Prospective, multicenter, double-masked, sham-controlled RCT (36 months) India	153 patients with IU or PU, PanU in ≥ 1 eye for ≥ 1 year and ≥ 2 recurrences of uveitis requiring ocular injections or systemic therapy within past 12 months	**Active**: 0.2 µg/day FAi (*n* = 101) **Control**: Sham injection + standard of care (*n* = 52)	**Proportion of patients with uveitis recurrence in study eye (6-months**,** 1-**,** 3-years) (%)**: FAi: 21.8, 32.7, 46.5; Control: 53.8, 59.6, 75.0 (*p* < 0.001 at 6 months, *p* = 0.002 at 1 year and *p* < 0.001 at 3 years) **Median time to first recurrence (days)**: FAi: 1116.0; Control: 190.5 **≥ 15-letter increase in BCVA from baseline at 36 months (%)**: FAi: 26.7; Control: 27.3 **Patients receiving at ≥ 1 adjunctive systemic medication (%)**: FAi: 31.7; Control: 32.7 **Patients receiving ≥ 1 local corticosteroid injection (%)**: FAi: 8.9; Control: 51.9	**IOP events > 25 mmHg**,** > 30 mmHg (%)**: FAi: 23.8, 16.8; Control: 3.8, 1.9 **Glaucoma surgery (%)**: FAi: 2.0; Control: 0 **Cataract surgery (%)**: FAi: 70.5; Control: 26.5 **Hypotony (< 6 mmHg) (%)**: FAi: 9.9; Control: 0
**Intravitreal dexamethasone implant**					
NCT00333814 (HURON), 2011 [17]	Prospective, multicenter, masked, parallel-group, sham-controlled RCT (26 weeks) United States, Australia, Austria, Brazil, Canada, Czech Republic, France, Germany, India, Israel, Republic of Korea, Poland, South Africa, Spain, Switzerland, United Kingdom	229 adult patients with IU or PU	**Active**: 0.7 mg DEXi (*n* = 77) **Active**: 0.35 mg DEXi (*n* = 76) **Control**: Sham procedure (*n* = 76)	**Proportion of eyes with VH grade 0 at week 8 (%)**: DEXi 0.7 mg: 47; DEXi 0.35 mg: 36; Control: 12 (*p* < 0.001) **Mean (SD) change from baseline in CRT: 8 weeks**,** 26 weeks (µm)**: DEXi 0.7 mg: -99.4 (151.8), -50.2 (102.9); DEXi 0.35 mg: -91.0 (132.8), -68.1 (138.8); Control: -12.4 (123.7), -35.5 (134.9) *p* ≤ 0.004 at 8 weeks, *p* ≥ 0.227 at week 26 **Percentage of eyes requiring rescue medication: 3 weeks**,** 26 weeks (%)**: DEXi 0.7 mg: 1, 22; DEXi 0.35 mg: 3, 25; Control: 15, 38 (*p* = 0.004) at 3 weeks, *p* = 0.030 at week 26	**Eyes with IOP ≥ 25 mmHg (%)**: DEXi 0.7 mg: 7.1; DEXi 0.35 mg: 8.7; Control: 4.2 (*p* > 0.05 at any visit) **Cataracts in phakic eyes (%)**: DEXi 0.7 mg: 15; DEXi 0.35 mg: 12; Control: 7 (*p* = 0.769)
**Suprachoroidal triamcinolone acetonide injection**					
NCT02595398 (PEACHTREE), 2020 [21]	Multicenter RCT (24 weeks) United States, India, Israel	160 patients with uveitic ME	**Active**: 4 mg SC-TCA (*n* = 96) **Control**: Sham procedure (*n* = 64) **Treatment administration**: Baseline and week 12	**Improvement from baseline ≥ 15 ETDRS letters in BCVA at week 24 (%)**: SC-TCA: 46.9; Sham: 15.6 (*p* < 0.001) **Reduction from baseline in CRT at week 24 (µm)**: SC-TCA: -153; Sham: -18 (*p* < 0.001) **Patients with ME resolution (CRT < 300 μm) at week 4 (%)**: SC-TCA: 53; Sham: 2 (*p* < 0.05) **Percentage of patients requiring rescue therapy (%)**: SC-TCA: 13.5; Sham: 72 (*p* < 0.001)	**Elevated IOP (%)**: SC-TCA: 11.5; Sham: 15.6 **Cataract (%)**: SC-TCA: 7.3; Sham: 6.3
NCT02952001 (MAGNOLIA), 2022 [22]	Multicenter, non-interventional RCT. Extension of PEACHTREE (24 weeks to 48 weeks) United States, India	33 patients who completed PEACHTREE with no rescue therapy	Prior to crossover: Previously received SC-TCA (*n* = 28) Prior to crossover: previously received sham (*n* = 5) **Treatment administration**: After crossover, no intervention	**Median time to rescue therapy (days)**: SC-TCA: 257; Sham: 55.5 **SC-TCA treated patients not requiring rescue therapy (%)**: 50% **Mean gain in BCVA in SC-TCA treated patients not rescued (ETDRS letters)**: Crossover visit (end of PEACHTREE): +16.8 (*p* < 0.001); 48 weeks: +12.1 (*p* < 0.004) **Mean CRT reduction in SC-TCA treated patients not rescued (µm)** **CRT**: Crossover visit (end of PEACHTREE): -178.1 (*p* < 0.001); 48 weeks: -174.5 (*p* < 0.001)	**IOP increase ≥ 10 mmHg (%)**: SC-TCA: 14.3; Sham: 0 **IOP ≥ 25 mmHg (%)**: SC-TCA: 7.1; Sham: 0 **Cataract (%)**: SC-TCA: 25.0; Sham: 20
**Intravitreal non-corticosteroid drug injection**					
NCT01846299 (PROMETHEUS), 2018 [25]	Multicenter, double-masked, sham-controlled RCT (12 months)	178 adult patients with ME from multiple causes including 21 with uveitis	IVR 0.5 mg (*n* = 118, including 14 with uveitis Sham intravitreal injection (*n* = 60, including 7 with uveitis Open-label IVR after month 2	**Uveitis cohort (*n*** **= 21)**: **Mean change (95% CI) from baseline at month 2 (letters)**: IVR: 6.2 (0.28–12.03); Sham: 1.0 (-5.43–7.43) **Mean change (95% CI) from baseline at month 12 (letters)**: IVR: 5.9 (-0.94–12.78); Sham: 10.0 (1.82–18.18) **Mean CRT change (95% CI) from baseline at month 2 (µm)**: IVR: -95.1 (-197.17–6.88); Sham: -51.4 (-194.82–91.97) **Mean CRT change (95% CI) from baseline at month 12 (µm)**: IVR: -94.1 (-172.65 – -15.52); Sham: -113.4 (-281.69–54.84) **Mean (SD) treatment exposure (injections)**: IVR: 7.1 (4.2); Sham: 5.8 (3.0)	**Full cohort (*n*** **= 178: IVR = 119**,** Sham with IVR = 56**,** Sham without IVR = 2)**: **Macular edema (%)**: IVR: 5.9; Sham with IVR: 3.6; Sham without IVR: 0 **IOP increase (%)**: IVR: 5; Sham with IVR: 5.4; Sham without IVR: 0 **VA reduced (%)**: IVR: 9.2; Sham with IVR: 5.4; Sham without IVR: 0
NCT01358266 (SAKURA 1 & 2), 2020 [27]	Multicenter, double-masked, active-controlled RCTs (6 months) United States, Argentina, Austria, Brazil, Chile, Colombia, France, Germany, India, Israel, Italy, Japan, Peru, Poland, Spain, Turkey, United Kingdom	592 adult patients with IU, PU or PanU	IVS 44 µg (active control) (*n* = 208) IVS 440 µg (*n* = 208) IVS 880 µg (*n* = 177) IVS 880 µg arm was discontinued after interim analysis showed no advantage over IVS 440 µg	**Patients with VH grade 0 at month 5**,** without rescue therapy (%)**: IVS 44 µg: 13.5; IVS 440 µg: 21.2 (*p* = 0.038) **Patients with VH grade 0 or 0.5 + at month 5 (%)**: IVS 44 µg: 40.4; IVS 440 µg: 50 (*p* = 0.017) **Corticosteroid-tapering at month 5 (%)**: IVS 44 µg: 68.8; IVS 440 µg: 69.6 (*p* = 0.94) **Patients with ≥ 5 letter improvement from baseline by month 5 (%)**: IVS 44 µg: 48.4; IVS 440 µg: 44.9 (*p* = 0.94) **Mean change in CRT at month 5 if macular edema at baseline (*n*** **= 133) (%)**: IVS 44 µg: -12.4; IVS 440 µg: 20.4 (*p* = 0.047)	**Worsening uveitis (%)**: IVS 44 µg: 6.3; IVS 440 µg: 6.8; IVS 880 µg: 9.6 **Sterile endophthalmitis (%)**: IVS 44 µg: 0; IVS 440 µg: 0.5; IVS 880 µg: 3.9 **Endophthalmitis (%)**: IVS 44 µg: 0; IVS 440 µg: 0.5; IVS 880 µg: 0 **Increased IOP (%)**:IVS 44 µg: 1; IVS 440 µg: 1.5; IVS 880 µg: 0.6 **Glaucoma surgery (%)**:IVS 44 µg: 1; IVS 440 µg: 2; IVS 880 µg: 1.7 **Cataract (%)**:IVS 44 µg: 1; IVS 440 µg: 0.5; IVS 880 µg: 2.2
**Comparisons of corticosteroid or non-corticosteroid therapeutics delivered locally to the eye**					
NCT02374060 (POINT), 2019 [28]	Multicenter, randomized, parallel-treatment, comparative trial (24 weeks) United States, Australia, Canada, United Kingdom	235 eyes of 192 patients with uveitic ME	PTA: 73 eyes of 65 patients IVTA: 79 eyes of 62 patients DEXi: 78 eyes of 63 patients	**CRT reduction relative to baseline at 8 weeks (%)**: PTA: 23; IVTA: 39; DEXi: 46 **IVTA and DEXi superior to PTA (HR**,** 99.87% CI)**: IVTA 0.79, 99.87 (0.65–0.96); DEXi 0.69, 99.87 (0.56–0.86) **DEXi non-inferior to IVTA at 8 weeks (HR**,** 99.87% CI)**: 0.88 (0.71–1.08) **BCVA**: Improved in all groups throughout follow-up at 4, 8, 12, 20, 24 weeks	**IOP ≥ 24 mmHg when compared to PTA (HR**,** 95% CI)**: higher for DEXi (2.52, 1.29–4.91), higher for IVTA (1.83, 0.91–3.65) **IOP elevation ≥ 24 mmHg**: Did not differ significantly between DEXi and IVTA **IOP elevation ≥ 30 mmHg (number)**: PTA: 4, DEXi: 3, ITA: 5 **IOP-lowering medications (%)**: Did not differ significantly between groups: Baseline: 22; 8 weeks: 32; 24 weeks: 39
NCT02623426 (MERIT), 2023 [29]	Multicenter, randomized, parallel-treatment, comparative effectiveness trial (24 weeks) United States, Australia, Canada, India, United Kingdom	225 eyes of 194 patients with uveitic ME	DEXi: 77 eyes of 65 patients IVM: 79 eyes of 65 patients IVR: 69 eyes of 64 patients	**CRT reduction relative to baseline at 12 weeks (%)**: DEXi: 35; IVM: 11; IVR: 22% **DEXi significantly more effective in reducing CRT**: IVM (*p* < 0.001), IVR (*p* < 0.018) **BCVA at 12 weeks**: BCVA gain greater for DEXi than IVM (+ 4.5 letters, *p* < 0.002) or IVR (+ 3.5 letters, *p* < 0.02)	**IOP ≥ 24 mmHg (% & HR**,** 95% CI vs. DEXi if*p*** **< 0.05)**: DEXi: 22.1; IVM: 2.5 & 0.10 (0.02–0.44); IVR: 5.9 & 0.25 (0.08–0.74) **Change in baseline IOP ≥ 10 mmHg (% & HR**,** 95% CI vs. DEXi if*p*** **< 0.05)**: DEXi: 12.9; IVM: 2.5 & 0.17 (0.04–0.80); IVR: 2.9 & 0.24 (0.05–1.12) **Increase in IOP-lowering medication (%)**: Did not differ significantly between groups: DEXi: 22.1; IVM: 7.6; IVR: 8.8 **Decrease in BCVA ≥ 15 letters (% & HR**,** 95% CI vs. DEXi if*p*** **< 0.05)**: DEXi: 5.2; IVM: 19.5 & 3.96 (1.34–11.66); IVR: 10.3

**Abbreviations**: BCVA: best-corrected visual acuity; CI: confidence interval; CRT: central retinal thickness; DEXi: intravitreal dexamethasone implant; ETDRS: Early Treatment Diabetic Retinopathy Study; FAi: intravitreal fluocinolone acetonide implant; HR: hazard ratio; IOP: intraocular pressure; IU: intermediate uveitis; IVM: intravitreal methotrexate; IVR: intravitreal ranibizumab; IVS: intravitreal sirolimus; IVTA: intravitreal triamcinolone acetonide; ME: macular edema; mo: months; NR: not reported; OCT: optical coherence tomography; PanU: panuveitis; PTA: periocular triamcinolone acetonide; PU: posterior uveitis; RCT: randomized controlled trial; SC-TCA: suprachoroidal triamcinolone acetonide; SD: standard deviation; TA: triamcinolone acetonide; VH: vitreous haze

### Intravitreal fluocinolone acetonide implant

A surgically placed intravitreal fluocinolone acetonide implant was the first sustained-release corticosteroid device to be approved for the treatment of non-infectious intermediate, posterior, and pan- uveitis, delivering the drug within the eye for up to approximately 36 months. Its safety and effectiveness were evaluated in four 36-month prospective phase III clinical trials [11–14]. Different concentrations and formulations of fluocinolone acetonide effectively controlled the intraocular inflammation, reducing recurrence rates, extending recurrence-free intervals, and decreasing the need for adjuvant therapy compared to controls.

#### NCT00407082

Surgically implanted intravitreal fluocinolone acetonide implants containing one of two doses of drug (0.59 mg and 2.1 mg) were compared in 278 patients with non-infectious uveitis involving the posterior segment for at least 12 months [11], with initial United States Food and Drug Administration (FDA) approval based on a 34-week interim analysis [15]. In patients with bilateral disease, the implant was placed in the eye with more severe inflammation. Rapid release of drug from the 2.1 mg implant, delivering higher-than-intended concentrations and risking early depletion before 36 months, prompted a mid-trial implant redesign. The uveitis recurrence rate after implantation was lower for the 0.59 mg implant, with recurrences dropping significantly from 62% pre-implantation to 4%, 10%, and 20% at 1, 2, and 3 years post-implantation, respectively. The 2.1 mg implant also reduced recurrences, but there was no additional benefit conferred from the higher dose. Eyes with implants had longer mean duration to first uveitis flare, and were more likely than fellow eyes to have a 15 or more Early Treatment Diabetic Retinopathy Study (ETDRS) letter increase in best-corrected visual acuity from baseline at 36 months. Implanted eyes demonstrated a nearly 80% reduction in the use of systemic immunomodulatory drugs, as well as a significant decrease in macular edema. However, by 3 years, 40% of implanted eyes required glaucoma surgery (versus 2% of fellow eyes), and 93% of phakic implanted eyes required cataract surgery (versus 20% of fellow phakic eyes). Additionally, 75% of implanted eyes had elevations of intraocular pressure over 9 mmHg.

#### NCT0456482

The two intravitreal fluocinolone acetonide implants (0.59 mg and 2.1 mg) were evaluated in 239 predominantly Asian patients with chronic, recurrent non-infectious uveitis involving the posterior segment [12], demonstrating similar efficacy in inflammation control to the NCT00407082 trial [11]. In 0.59 mg implant eyes, uveitis recurrence dropped from 44% pre-implantation to 13%, 14%, and 17% at 1, 2, and 3 years post-implantation, respectively. The 2.1 mg implant offered similar benefits initially, but showed higher recurrence by the third year, indicating faster drug depletion. In contrast, fellow non-implanted eyes saw recurrence rates rise significantly from 20% in the year pre-implantation to 60% by 36 months. Eyes with implants required fewer periocular and systemic adjunctive treatments, and were significantly more likely to gain 3 or more logarithm of the minimum angle of resolution (logMAR) lines of best-corrected visual acuity. The mean area of macular edema in eyes treated with the 0.59 mg implant decreased by approximately 4-fold from screening to week 34, and this benefit was sustained over the 36-month study period. Cataract surgery was required in over 90% of phakic implanted eyes, and approximately 70% experienced intraocular pressure elevations to 10 mmHg or higher.

#### NCT01694186

A low dose injectable intravitreal fluocinolone acetonide implant or insert (0.19 mg, releasing 0.2 µg/day) was assessed in a clinical trial involving 129 patients with posterior segment non-infectious uveitis who were randomized 2:1 to receive an implant or a sham injection and standard of care [13]. The implant significantly reduced cumulative uveitis recurrences and extended median time to first recurrence compared to control (657 days versus 70.5 days). Fewer implanted eyes required adjunctive topical, locally injected and/or systemic anti-inflammatory treatments (58% versus 98%). At month 36, investigator-determined macular edema was observed in less eyes in the implant-treated group compared to sham (13% versus 27%), although the difference was not statistically significant. Pressure-lowering surgeries were more common with the implant (12% versus 6%), but were less common than with higher-dose implants. Nearly 75% of phakic implanted eyes needed cataract surgery by 36 months.

#### NCT02746991

In a clinical trial conducted in India of the low dose implant (0.19 mg) in 153 patients with posterior segment non-infectious uveitis, randomized to implant or sham injection plus standard of care, the implant cohort experienced better inflammation control with lower recurrence rates, extended time to recurrence, more vitreous haze resolution, and reduced need for adjunctive local corticosteroid injections [14]. Use of systemic immunomodulatory treatments was similar across cohorts. At month 36, fewer eyes in the implant-treated group had macular edema than in the sham group (24% versus 46%). An intraocular pressure over 25 mmHg was recorded for nearly one-quarter of implant eyes versus less than 5% of sham eyes, and cataract surgery was also more common in implant eyes (71% versus 27%).

### Intravitreal dexamethasone implant

The injected intravitreal dexamethasone implant (0.7 mg) provides sustained-release of drug to the posterior segment, and is commonly used either as monotherapy or alongside systemic immunomodulatory therapies for non-infectious intermediate, posterior, and pan- uveitis. It received FDA approval in 2010, based on the results of the HURON study [16], which demonstrated its efficacy in controlling ocular inflammation and improving visual acuity in patients with non-infectious intermediate or posterior uveitis [17, 18]. A recent observational study showed consistent benefit in real-world use for patients with non-infectious uveitis involving the posterior eye [18].

#### NCT00333814 (HURON)

The HURON study evaluated two intravitreal dexamethasone implants (0.7 mg and 0.35 mg) versus a sham procedure over 6 months for non-infectious intermediate or posterior uveitis [17]. A significant majority of the 229 enrolled patients − 81% – had intermediate uveitis. Patients were randomized 1:1:1 to receive the 0.7 mg or 0.35 mg implant, or a sham procedure in one eye. At week 8 post-treatment, there was a significant difference in the percentage of patients with grade 0 vitreous haze across the groups: this primary outcome was achieved by 47%, 36%, and 12% of eyes in the 0.7 mg implant, 0.35 mg implant, and sham groups, respectively. Improvement in vitreous haze was observed as early as 3 weeks following treatment. The dexamethasone implant groups also more often had improvement of 15 or more ETDRS letters in best-corrected visual acuity, plus less need for rescue medication. Central retinal thickness reduction was significantly better in both dexamethasone implant cohorts versus sham at week 8, but not week 26. Intraocular pressure elevations over 24 mmHg were observed in 7% of eyes that received the 0.7 mg dexamethasone implant, 9% that received the 0.35 mg dexamethasone implant, and 4% that had the sham treatment, with no significant difference between the groups. While no eyes required incisional glaucoma surgery, 23% of the 0.7 mg implant cohort required medication to lower the intraocular pressure. Cataract rates did not differ significantly between groups through week 26.

### Suprachoroidal triamcinolone acetonide injection

Drug placed in the suprachoroidal space targets the chorioretinal tissues while limiting anterior segment exposure, potentially lowering adverse events involving the anterior eye [19]. A triamcinolone acetonide suspension delivered via suprachoroidal microinjector received FDA approval for macular edema associated with non-infectious uveitis in 2021 [20]. Approval was based on the PEACHTREE study [21], which was extended as the MAGNOLIA study [22]. These clinical trials demonstrated significant improvements in central retinal thickness and best-corrected visual acuity after treatment with the triamcinolone acetonide therapeutic.

#### NCT02595398 (PEACHTREE)

The PEACHTREE trial was a 24-week, sham-controlled study that randomized 160 patients with uveitic macular edema 3:2 to unilateral suprachoroidal injection of triamcinolone acetonide (4.0 mg) or a sham procedure [21]. Patients received treatments at day 0 and week 12. The primary outcome of improvement of 15 ETDRS letters or more in best-corrected visual acuity at week 24 was achieved by significantly more patients in the treatment group than the sham group (47% versus 16%). Suprachoroidal triamcinolone acetonide produced greater reduction in central retinal thickness (153 μm versus 18 μm), with edema resolution rates of 53% at week 4 and 55% at week 24, compared to 2% and 12% for the sham procedure. Post-procedure intraocular pressure elevations were not significantly different between groups. Post-hoc analyses confirmed the effectiveness of the suprachoroidal triamcinolone acetonide injection across all anatomical subtypes of non-infectious uveitis [23]. Another post-hoc analysis highlighted potential benefit for patients on baseline systemic corticosteroids or corticosteroid-sparing therapies, who tended to have more severe disease, with similar need for rescue therapy across the groups [24].

#### NCT02952001 (MAGNOLIA)

The MAGNOLIA trial was an observational extension of PEACHTREE that evaluated longer term efficacy and safety of suprachoroidal triamcinolone acetonide by assessing time to rescue therapy over an additional 24 weeks in 28 treated and 5 control patients [22]. Among those not requiring rescue during PEACHTREE, the median time to rescue was 257 days for treatment versus 55.5 days for sham. One-half of the treated patients avoided rescue therapy for approximately 9 months, with an average gain of 12 ETDRS letters in best-corrected visual acuity and a mean 175 μm reduction in central retinal thickness at week 48. The extension showed that 14% of treated patients experienced intraocular pressure increases over 10 mmHg, and 11% required pressure-lowering medication, while cataract formation or progression occurred in 11%.

### Intravitreal non-corticosteroid drug injection

#### NCT01846299 (PROMETHEUS)

The PROMETHEUS trial evaluated intravitreal ranibizumab injection (0.5 mg) in adult patients with persistent macular edema from uncommon causes in a 12-month, sham-controlled clinical trial of 178 patients [25]. A subgroup analysis for uveitic macular edema included 14 patients treated with intravitreal ranibizumab and 7 patients who had a sham injection. Participants received baseline treatment, then pro re nata (PRN) dosing of their assigned treatment at 1 month based on disease activity, with open-label PRN intravitreal ranibizumab available to both groups from 2 months. Rescue options, including laser photocoagulation and periocular corticosteroid injections, were permitted at 1 month. The primary outcome was mean change in best-corrected visual acuity 2 months after the first treatment. For the uveitic macular edema subset, this was an almost 6 ETDRS letter increase in the ranibizumab-treated group and 1 in the sham group, not differing significantly, but likely related to the very small number of patient with uveitis; the 12-month change in best-corrected visual acuity was also non-significant between the groups. Ranibizumab was well tolerated, with no safety concerns beyond those described for the standard indications.

#### NCT01358266 (SAKURA)

The SAKURA trials 1 and 2 assessed the efficacy and safety of intravitreal sirolimus injection in non-infectious uveitis active in the posterior segment as two international randomized, double-masked 6-month studies [26]. A combined analysis of 592 patients was published [27]. Participants were randomized 1:1:1 to treatment with an intravitreal injection of sirolimus dosed at 44 µg, 440 µg, or 880 µg on days 1, 60, and 120. The 880 µg dose was discontinued after an interim analysis showed no advantage over the 440 µg dose. At month 5, the 440 µg dose significantly reduced ocular inflammation and achieved a vitreous haze score of 0 without rescue therapy in 21.2% of patients, compared to 13.5% with the 44 µg dose. Approximately 80% of patients in both these groups maintained or improved best-corrected visual acuity by 5 or more ETDRS letters, with no effect on intraocular pressure.

### Comparisons of corticosteroid or non-corticosteroid therapeutics delivered locally to the eye

#### NCT02374060 (POINT)

The POINT randomized clinical trial enrolled 235 eyes of 192 patients to compare the effectiveness of three locally injected corticosteroid treatments for uveitic macular edema: periocular triamcinolone acetonide injection (40 mg, periorbital floor or posterior sub-Tenon’s approach), intravitreal triamcinolone acetonide injection (4 mg), and intravitreal dexamethasone implant (0.7 mg) [28]. Both intravitreal treatments were superior to the periocular treatments in improving best-corrected logMAR visual acuity and reducing central retinal thickness. At 6 months, the two intravitreal therapeutics resulted in an approximate gain of 9 letters, compared to 4 for the periocular treatment. Central retinal thickness was reduced at 8 weeks in 39%, 46%, and 23% for intravitreal triamcinolone acetonide, intravitreal dexamethasone, and periocular triamcinolone acetonide, respectively. Intraocular pressure elevation was similar between the two intravitreal treatments, with up to one-third of patients requiring pressure-lowering medications by 6 months, and was significantly more likely than after the periocular injection.

#### NCT02623426 (MERIT)

The MERIT trial evaluated the effectiveness of three injected intravitreal treatments for uveitic macular edema: dexamethasone implant (0.7 mg), methotrexate injection (0.4 mg), and intravitreal ranibizumab (0.5 mg) [29]. A total of 194 participants with minimally active or inactive uveitis and persistent or recurrent macular edema were randomized to 1:1:1 to one treatment to one or both eyes depending on laterality of the disease. The dexamethasone implant gave the best results at 12 weeks, with a 35% reduction in central retinal thickness versus 11% for methotrexate and 22% for ranibizumab. Only the dexamethasone implant achieved a significant improvement in best-corrected visual acuity of almost 5 letters, while methotrexate injection was associated with the highest occurrence of vision loss, mostly related to persistent macular edema. Elevations of the intraocular pressure to 24 mmHg or higher occurred in 22% of the dexamethasone implant group, which was significantly more frequent than in the methotrexate and ranibizumab groups. However, pressure spikes above 30 mmHg were measured equally across the treatment groups.

## Phase III clinical trials of systemic immunomodulatory drugs for non-infectious uveitis

In clinical practice, a wide range of systemic immunomodulatory drugs are used to manage non-infectious uveitis. However, only a handful of these drugs–methotrexate, mycophenolate mofetil, interferon-beta, adalimumab, gevokizumab, and secukinumab – have been evaluated in completed and published phase III clinical trials. These clinical trials are described in this section. Table 2 presents a summary of the trial design, including drug dosing schedules and outcome measures, along with the key findings.

**Table 2:** Summary of phase III clinical trials of systemic immunomodulatory drugs for non-infectious uveitis

Identification number (name), year [Ref]	Design (duration) and location	Participants and study arms	Uveitis subtype	Outcome measures*	Key effectiveness and safety findings
NCT01232920, 2014 [31]	Multicenter, randomized, observer-masked clinical trial (6 months) India	*n* = 80: 41 methotrexate (25 mg, PO, weekly) vs. 39 mycophenolate mofetil (2 g, PO, daily)	IU, PU, PanU	**Primary endpoint**: Uveitis control with ≤ 10 mg/day of prednisone and ≤ 2 drops/day of prednisolone acetate 1%, without treatment failure due to AEs at 5 and 6 months. **Secondary endpoints**: Time to corticosteroid-sparing control of inflammation; change in BCVA; resolution of macular edema; frequency of AEs; treatment adherence; treatment success by anatomic location.	69% of methotrexate and 47% of mycophenolate group achieved uveitis control (*p* = 0.09). No significant differences in macular edema resolution (77% in methotrexate group and 54% in mycophenolate group) and other secondary outcomes. AEs did not significantly differ between groups.
NCT01829295 (FAST), 2019 [30]	Multicenter, randomized, observer-masked clinical trial (12 months) United States, Australia, India, Mexico, Saudi Arabia	*n* = 216: 107 methotrexate (25 mg, PO, weekly) vs. 109 mycophenolate mofetil (3 g, PO, daily)	Active IU, PU, PanU	**Primary endpoint**: Uveitis control at 6 months (≤ 0.5 + anterior chamber cells; ≤ 0.5 + VH; inactive retinal/choroidal lesions) with ≤ 7.5 mg/day of oral prednisone and ≤ 2 drops/day of prednisolone acetate 1%, without treatment failure due to intolerability or safety. **Secondary endpoints**: Uveitis control by uveitis subtype and by enrollment site; uveitis control at 12 months; change in BCVA; central retinal thickness at 6 months.	67% of methotrexate group and 57% mycophenolate group achieved uveitis control (*p* = 0.2). Patients with PU and panuveitis had greater control of uveitis with methotrexate (74.4% vs. 55.3%; *p* = 0.02). Response was maintained at 12 months in 80% of methotrexate group and 74% of mycophenolate group. No significant differences in BCVA and central retinal thickness across treatments. Increased liver enzymes in 13% of methotrexate group vs. 7.4% of mycophenolate group.
NCT00344253, 2013 [33]	Monocentric, randomized clinical trial (3 months) Germany	*n* = 19: 9 interferon-beta (44 mg, SC, 3 times/week) vs. 10 methotrexate (20 mg, SC, weekly)	IU	**Primary endpoint**: Mean change in BCVA at 3 months. **Secondary endpoints**: Improvement of macular edema on OCT; AC cells and VH grade; rate of ocular complications; quality of life.	Interferon-beta led to a greater improvement in BCVA compared with methotrexate (mean improvement of 0.31 logMAR vs. 0.09; *p* = 0.004). Decrease in macular edema in 100% of patients on interferon-beta vs. 25% on methotrexate (*p* = 0.002). Interferon-beta had more AEs than methotrexate.
NCT01138657 (VISUAL I), 2016 [34]	Multicenter, double-masked, randomized, placebo-controlled trial (until treatment failure, or completion of 80 weeks of treatment, or when 138 treatment-failure events occurred) United States, Canada, Europe, Israel, Australia, Latin America	*n* = 217: 110 adalimumab (loading dose of 80 mg, SC, followed by 40 mg biweekly) vs. 107 placebo	Active IU, PU, PanU	**Primary endpoint**: Time to treatment failure at or after 6 weeks. **Secondary endpoints**: Ranked: (1) change in AC cell grade; (2) change in VH grade; (3) change in BCVA; (4) time to macular edema onset; (5) percentage change in central retinal thickness; (6–9) change in VFQ-25 scores.	Time to treatment failure was 24 weeks for adalimumab vs. 13 weeks for placebo (HR 0.5; *p* < 0.001). Adalimumab was effective for idiopathic uveitis (*p* = 0.003) and IMT-naïve patients at baseline (*p* < 0.001), but not for birdshot choroidopathy (*p* = 0.09) or those previously on IMT (*p* = 0.05). Worsening of AC cell and VH grade and decrease in BCVA were less frequent in the adalimumab group (*p* ≤ 0.01). In those without baseline macular edema, macular holes, or retinal detachment, the risk of macular edema was reduced in 67% with adalimumab (*p* = 0.02). Change in most of VFQ-25 scores favored adalimumab group. AEs:1052 events/100 PY in the adalimumab group and 972 events/100 PY in the placebo group; SAEs: 29 events/100 PY in the adalimumab group and 14 events /100 PY in the placebo group.
NCT01124838 (VISUAL II), 2016 [35]	Multicenter, double-masked, randomized, placebo-controlled trial (until treatment failure at or after 2 weeks, or completion of 80 weeks of treatment, or when 106 treatment failures occurred) United States, Canada, Europe, Israel, Australia, Latin America	*n* = 229: 115 adalimumab (loading dose of 80 mg, SC, followed by 40 mg biweekly) vs. 114 placebo	Inactive IU, PU, PanU	**Primary endpoint**: Time to treatment failure in at least one eye. **Secondary endpoints**: Ranked: (1) change in AC cell grade; (2) change in VH grade; (3) change in BCVA; (4) time to macular edema onset; (5) percentage change in central retinal thickness; (6–9) change in VFQ-25 scores.	Time to treatment failure was delayed in the adalimumab group (median time not estimated [> 18 months] vs. 8.3 months with placebo, HR 0·57; *p* = 0.004). Treatment failure reported in 39% on adalimumab vs. 55% on placebo (median follow-up = 245 days). Testing of secondary endpoints was stopped after the first endpoint did not significantly differ from placebo. AEs did not significantly differ between groups.
NCT01148225 (VISUAL III), 2021 [39]	Multicenter, open-label extension study of VISUAL I and II (Efficacy results through 150 weeks and safety results up to 362 weeks of treatment) Argentina, Australia, Austria, Belgium, Brazil, Canada, Czech Republic, Denmark, France, Germany, Greece, Israel, Italy, Japan, Mexico, Poland, Portugal, Spain, Switzerland, United Kingdom, United States	*n* = 424 210 from VISUAL I & 241 from VISUAL II adalimumab (40 mg, SC, biweekly)	IU, PU, PanU	**Primary endpoint**: Quiescence at week 150 in both eyes. **Secondary endpoints**: Macular edema; proportion of patients without worsening of BCVA ≥ 15 letters on ETDRS chart; dose of systemic corticosteroids and IMT.	80% of patients with active and 96% with inactive uveitis at study entry were quiescent at 150 weeks. 54% of the active uveitis group and 89% of the inactive uveitis group achieved corticosteroid-free quiescence. Systemic corticosteroids use was reduced in all patients (9.4 mg ± 17.1 to 1.5 mg ± 3.9). IMT had a mean dose reduction of -36% and − 29% in patients with active and inactive uveitis at study entry, respectively. AEs: 396 events/100 PY; SAEs: 15 events/100 PY.
EudraCT number: 2010-021141-41 (SYCAMORE), 2017 [36]	Multicenter, double-masked, randomized, placebo-controlled trial (Until treatment failure or completion of 18 months of treatment) United Kingdom	*n* = 90: 60 adalimumab (20 mg, SC, if < 30 kg or 40 mg if ≥ 30 kg, biweekly) + methotrexate (10–20 mg/m2, PO or SC, weekly; maximum dose of 25 mg) vs. 30 placebo + methotrexate	Active JIA-associated uveitis	**Primary endpoint: T**ime to treatment failure. **Secondary endpoints**: Therapy tolerability; use of corticosteroids (topical and systemic); change in BCVA; resolution of macular edema.	Time to treatment failure was delayed by adalimumab (median time not reached with adalimumab and of 24.1 weeks with placebo; HR 0.25, 95% CI 0.12 − 0.49, *p* < 0.0001). Treatment failure in 27% of adalimumab and 60% in placebo group (*p* = 0.002). More patients on adalimumab could reduce topical corticosteroids to < 2 drops/day. Systemic use of corticosteroids and change in BCVA were similar between groups. AEs were more common with adalimumab.
NCT01385826 (ADJUVITE), 2018 [42]	Multicenter, double-masked, randomized, placebo-controlled trial (2 months double-masked phase and 10 months open-label phase) France	*n* = 32: 16 adalimumab (24 mg/m2, SC, in patients < 13 years, 40 mg in the others) vs. 16 placebo	Active JIA-associated uveitis	**Primary endpoint**: ≥ 30% reduction of inflammation on laser flare photometry, and no worsening of cell count or protein flare on slit lamp examination at 2 months.	≥ 30% reduction in intraocular inflammation in 56% of adalimumab vs. 20% of placebo group. Non-SAEs were similar between groups. One SAE reported during the double-masked phase in the placebo group, and 6 SAEs in the open-labelled phase. No SAEs associated with adalimumab.
NCT01965145 (EYEGUARD B), 2018 [43]	Two-center, double-masked, randomized, placebo-controlled trial (Time to first ocular exacerbation, ending when predefined number of exacerbations occurred in core study, followed by 1-year extension period United Kingdom, South Korea	*n* = 83: 40 gevokizumab (60 mg, SC, every 4 weeks) vs. 43 placebo	Behçet’s disease-associated uveitis	**Primary endpoint**: Time to first ocular exacerbation. **Secondary endpoints**: Number of ocular flares; change in BCVA; AC cells and VH grade; macular thickness; FFA score; daily corticosteroid dose; rescue therapy; onset of macular edema.	Gevokizumab did not significantly reduce time to first ocular exacerbation (35% of uveitis flare-up in both groups). BCVA loss ≥ 10 letters ETDRS in 10% of gevokizumab vs. 26.8% of placebo group (OR = 0.29, *p* = 0.048). Gevokizumab reduced incidence of macular edema (10% vs. 18%), retinal infiltrates (21% vs. 29%), and retinal vasculitis (10% vs. 27%). AEs did not significantly differ between groups.
NCT00995709 (SHIELD), 2013 [44]	Multicenter, double-masked, randomized, placebo-controlled trial (24 weeks) United States, Egypt, France, Germany, Greece, Hong Kong, India, Israel, Italy, Jordan, Singapore, South Korea, Spain, Switzerland, Taiwan, Tunisia, Turkey	*n* = 118: 39 secukinumab 300 mg, SC, at baseline, week 1, and week 2 (loading phase); then every 2 weeks vs. 40 secukinumab 300 mg SC at baseline and week 2 (loading phase); then monthly vs. 39 placebo	Behçet’s disease-associated PU, PanU	**Primary endpoint: **Recurrence rate of uveitis at 24 weeks. **Secondary endpoints**: Change in IMT score; change in BCVA, VH score; safety outcomes.	Rate of ocular exacerbations over 24 weeks did not significantly differ between secukinumab group and placebo group. No differences in BCVA change and VH score. Secukinumab group had a reduction in mean IMT score. AEs were more common with secukinumab.
NCT01095250 (INSURE), 2013 [44]	Multicenter, double-masked, randomized, placebo-controlled trial (28 weeks) United States, Canada, Egypt, France, Germany, Hungary, India, Israel, Japan, Singapore, Spain, Switzerland, United Kingdom	*n* = 31: 8 secukinumab 300 mg, SC, at baseline, week 1, and week 2 (loading phase); then every 2 weeks vs. 10 secukinumab 300 mg SC at baseline and week 2 (loading phase); then monthly vs. 8 secukinumab 150 mg, SC, at baseline and week 2 (loading phase); then monthly vs. 5 placebo	Active non-Behçet’s IU, PU, PanU	**Primary endpoint**: Mean change in VH score from baseline to week 28 or at rescue therapy, if earlier. **Secondary endpoints**: Change in IMT score; change in BCVA, VH score; safety outcomes.	The study was terminated early due to failure to meet the primary endpoint.
NCT01032915 (ENDURE), 2013 [44]	Multicenter, double-masked, randomized, placebo-controlled trial (24 weeks) United States, Brazil, Germany, India, Israel, Italy, Spain, Switzerland, Turkey, United Kingdom	*n* = 125: 29 secukinumab 300 mg, SC, at baseline, week 1, and week 2 (loading phase); then every 2 weeks vs. 31 secukinumab 300 mg SC at baseline and week 2 (loading phase); then monthly vs. 31 secukinumab 150 mg, SC, at baseline and week 2 (loading phase); then monthly vs. 34 placebo	Inactive non-Behçet’s IU, PU, PanU	**Primary endpoint**: Time to first recurrence over 24 weeks. **Secondary endpoints**: Change in IMT score; change in BCVA, VH score; safety outcomes.	Results of an interim analysis did not show significant effects for any of the 3 secukinumab groups compared with placebo, therefore the study was discontinued. AEs were more common with secukinumab.

**Abbreviations**: AC: anterior chamber; AE: adverse event; BCVA: best-corrected visual acuity; CI: confidence interval; ETDRS: Early Treatment Diabetic Retinopathy Study, FFA: fundus fluorescein angiography; HR: hazard ratio; IU: intermediate uveitis; IOP: intraocular pressure; IMT: immunomodulatory therapy; JIA: juvenile idiopathic arthritis; logMAR: logarithm of the minimal angle of resolution; VFQ-25: 25-item National Eye Institute visual function questionnaire 25; OCT: optical coherence tomography; OR: odds ratio; PanU: panuveitis; PO: orally; PU: posterior uveitis; PY: patients-years; SAE: serious adverse event; SC: subcutaneously; VH: vitreous haze

*The definition of uveitis control varies depending on the study and typically is defined by ≤ 0.5 + AC cells, ≤ 0.5 + vitreous cells, ≤ 0.5 + VH, and inactive retinal or choroidal lesions. Treatment failure is defined by several criteria, most frequently including a 2-step increase in AC cells, vitreous cells or VH, persistent inflammation, new inflammatory chorioretinal or retinal vascular lesions, worsening of BCVA by ≥ 15 letters on ETDRS. Each study should be consulted for the specific criteria applied

### Antimetabolite drugs

Antimetabolite drugs have not been evaluated for the treatment of non-infectious uveitis in placebo-controlled, randomized clinical trials. However, methotrexate and mycophenolate mofetil were compared directly for effectiveness and safety in patients with non-infectious uveitis in the FAST study [30], as well a smaller initial clinical trial [31].

#### NCT01232920

Methotrexate was compared against mycophenolate mofetil for treating active non-infectious intermediate, posterior and pan- uveitis in a randomized clinical trial that enrolled 80 patients whose disease was immunosuppression-naïve and/or corticosteroid-resistant [31]. After 5 to 6 months, 69% of patients treated with methotrexate and 47% of patients treated with mycophenolate mofetil achieved the primary endpoint of controlled inflammation with corticosteroid-sparing. The 22% difference favoring methotrexate was not statistically significant, potentially due to underpowering of the study. Resolution of macular edema, which was a secondary endpoint, was achieved by 77% and 54% of patients in the methotrexate and mycophenolate mofetil treatment groups, respectively. Most adverse reactions were mild or moderate, with 10% of each group experiencing gastrointestinal symptoms, and another 10% showing altered hemoglobin levels or liver function tests, with no significant difference between groups. Six of the 80 patients had treatment failure due to intolerability or safety concerns, including four on methotrexate and two on mycophenolate.

#### NCT01829295 (FAST)

With a similar protocol as used in NCT01232920, FAST was a larger randomized clinical trial that enrolled 216 subjects with intermediate, posterior, and pan- uveitis [30]. This trial showed no significant difference between methotrexate and mycophenolate mofetil in achieving uveitis control at 6 months (67% with methotrexate versus 57% with mycophenolate mofetil). Patients with posterior and pan- uveitis benefited significantly more from methotrexate (74% versus 55% control), while the percentage success was similar for the two antimetabolites in patients with intermediate uveitis. Median visual acuity and central retinal thickness were similar between treatments. During the subsequent 6 months, when a drug-switch was permitted, 80% of methotrexate-treated and 74% of mycophenolate mofetil-treated patients maintained their positive responses. Patients switching from mycophenolate mofetil to methotrexate had superior responses, with over two-thirds achieving uveitis control, compared to just over one-third switching from methotrexate to mycophenolate mofetil. Most adverse events were mild or moderate, with fatigue and headache reported in around one-half of participants. Moderate elevation of liver enzymes was reported by 13% and 7% of methotrexate and mycophenolate mofetil users, respectively, whilst extremely raised liver enzymes were observed in less than 3% with each drug. Treatment failure due to poor tolerability and safety issues occurred in 9% of the methotrexate group and 5% of the mycophenolate mofetil group.

The FAST trial participants who had macular edema at baseline were the subject of a subanalysis [32]. At enrolment, 71 of the 194 patients (37%) who completed the primary outcome visit had macular edema. At 12 months, improvement in macular edema was observed in 42% of the methotrexate group and 60% of the mycophenolate mofetil group. For those individuals who switched their antimetabolite drug at 6 months, resolution occurred in 47% on methotrexate and 55% on mycophenolate mofetil. Overall, improvement and resolution rates were similar between the treatment groups. However, macular edema persisted in about half of the patients, even among those deemed a treatment success according to the primary outcome definition. Corticosteroid injections were used similarly in both groups, and visual acuity improvements were comparable.

### Interferon

#### NCT00344253

Interferon-beta was compared against methotrexate for idiopathic and multiple sclerosis-associated intermediate uveitis in a small randomized controlled clinical trial that showed superiority of interferon-beta in improving visual acuity and reducing macular edema after 3 months of treatment [33]. The trial was stopped early, given the superiority of the outcomes in the interferon-beta arm, resulting in only 19 participants in total being enrolled. All patients treated with interferon-beta experienced a reduction in macular edema by optical coherence tomography, compared with only one-quarter of the methotrexate group, a difference which achieved high statistical significance. Three-quarters of the methotrexate group switched to interferon-beta after 3 months due to lack of response, showing vision improvement and reduction of macular edema and inflammation at 1-year of follow-up. Interferon-beta was associated with almost twice as many adverse events as methotrexate, with flu-like symptoms being the most common reactions in the interferon-beta group and nausea in the methotrexate group.

### Adalimumab

The approval of adalimumab, a fully human monoclonal anti-tumor necrosis factor-alpha antibody, for the treatment of non-infectious uveitis by the regulatory agencies in the United States, Europe and beyond, was based on the phase III VISUAL I and II, and SYCAMORE clinical trials [34–36].

#### NCT01138657 (VISUAL I)

The VISUAL I trial involved 217 adults with corticosteroid-resistant intermediate, posterior, and pan- uveitis, randomized to treatment with adalimumab or placebo until a prespecified number of treatment-failure events had occurred [34]. Adalimumab significantly reduced the risk of treatment failure by 50% compared to placebo, with a median time to treatment failure of 24 weeks versus 13 weeks for placebo. An exploratory analysis showed that adalimumab was effective for idiopathic uveitis and patients not on immunomodulatory drugs at baseline, but not for birdshot chorioretinopathy and those patients already using immunomodulatory therapy. Time to development of macular edema did not differ between groups. However, among patients with no baseline macular edema, macular holes, or retinal detachment, the risk of developing macular edema was 67% lower in the adalimumab-treated group compared with placebo. Serious adverse reactions were reported as 28.8 events/100 patient-years in the adalimumab group, including tuberculosis and other respiratory infections, systemic lupus erythematosus, demyelinating disorders, and cancer, while the placebo group had 13.6 events/100 patients-years. Adverse reactions that led to discontinuation of treatment were also more common in the adalimumab group.

#### NCT01124838 (VISUAL II)

The VISUAL II trial assessed the efficacy of adalimumab in delaying treatment failure in patients with quiescent non-infectious intermediate, posterior and pan- uveitis, enrolling 229 subjects [35]. Treatment with adalimumab significantly delayed time to uveitis relapse by 43% compared to placebo, with failure occurring after 18 months, and over one-half of patients not experiencing relapse after a median follow-up of 245 days. In the placebo group, the median time to treatment failure was around 8 months. Adverse events led to the discontinuation of 14 and 16 members of the adalimumab and placebo groups, respectively. No significant differences in adverse reactions were noted between groups during follow-up. The incidence of adverse events was similar between treatment groups. Serious adverse events were reported at rates of 13.8 and 14.1 events per 100 patient-years in the adalimumab group and placebo group, respectively. One death due to aortic dissection and cardiac tamponade, one malignancy, and three cases of latent tuberculosis were reported in the adalimumab group, while the placebo group had one patient with latent tuberculosis. No active tuberculosis, systemic lupus erythematosus or lupus-like reactions, or demyelinating disorders were reported. Overall, 9% of the adalimumab group and 6% of the placebo group discontinued the study intervention due to adverse reactions, and 6% developed anti-adalimumab antibodies. A post-hoc assessment of data from both VISUAL I and VISUAL II studies showed overall higher scores of vision-related quality of life for adalimumab-treated patients than those in the placebo group (NEI VFQ-25 scores) [37].

A separate analysis of the Japanese populations in the VISUAL I and II trials was published [38]. This demonstrated that time to treatment failure with adalimumab was shorter for Japanese participants than other participants, with a non-significant difference between treatment and placebo groups. This may have been due in part to the small sample of Japanese participants (*n* = 16 in VISUAL I and *n* = 32 in VISUAL II), but additionally, the majority had panuveitis, which had the highest rate of treatment failure in these studies. Also, in VISUAL I, there was a Japanese participant outlier in the placebo group, who did not have treatment failure and thus impacted the study endpoints.

#### NCT01148225 (VISUAL III)

The VISUAL III study was an open-label extension of VISUAL I and II for 150 weeks (evaluating efficacy) and up to 362 weeks (evaluating safety) [39]. Of the 424 patients enrolled, 60 were excluded mostly due to missing data or compliance, or for requiring ocular surgery. At study start, 67% of patients had active uveitis and 33% of patients had quiescent uveitis. By week 150, 80% of those with active uveitis achieved quiescence, and 96% of those with quiescent uveitis maintained inactivity. At week 150, 54% and 89% of the patients whose uveitis was active and inactive at enrolment, respectively, enjoyed corticosteroid-free quiescence. Despite 46% of the patients in the active group requiring ongoing treatment with prednisone to maintain control of the uveitis, most were using no more than 7.5 mg/day. 68% of patients with active uveitis at the start of the study experienced flare(s) by the final visit, leading to a 9% discontinuation rate for adalimumab, and 39% of patients with initially inactive uveitis had recurrence(s), with 1% withdrawing. Cessation of additional immunomodulatory drugs was possible for 46% of the active uveitis group, and another one-third were able to lower the doses of these drugs. During up to 7 years of follow-up (median of 2.8 years), most adverse events were mild or moderate. Serious adverse events occurred in 24% of patients during the safety extension period, and 7% had severe adverse events at least possibly caused by adalimumab. There were 1.8 tuberculosis and 0.53 demyelinating disorder events per 100 person-years. Malignancies were reported in 3% of patients but were not attributed to adalimumab, except for non-melanoma skin cancer. Three of four deaths were considered unrelated to the drug. The drug was discontinued due to adverse reactions in 18% of patients. Interim results of VISUAL III from weeks 0 through 78 have also been detailed [40].

#### EudraCT number 2010-021141-41 (SYCAMORE)

The SYCAMORE trial compared the efficacy of adalimumab plus methotrexate to placebo plus methotrexate in delaying treatment failure in children and adolescents suffering from juvenile idiopathic arthritis (JIA)-associated uveitis [36]. Sixty patients received adalimumab, and 30 received placebo, with 15% and 23% of discontinuation, respectively, for reasons other than treatment failure. Over 18 months, the adalimumab plus methotrexate treatment group had a significantly longer time to treatment failure, amounting to a 75% risk reduction compared to the placebo plus methotrexate group. Treatment failure occurred in 27% of the adalimumab group versus 60% of the placebo group. However, adverse reactions were higher in the adalimumab group (10.07 versus. 6.51 events per patient-year), including severe reactions (22% versus 7%), of which infection was the most common. The study did not run sufficiently long to assess the incidence of cancer and demyelinating disease. The trial was unmasked at the second interim analysis due to the significant benefit of adalimumab, and the placebo group was moved to follow-up, while the adalimumab group continued treatment per the trial protocol.

For most SYCAMORE trial participants, the remission of uveitis induced by adalimumab did not last in the 1 to 2 years after discontinuation. Five-year follow-up of 28 children from a single center, including 19 in the adalimumab plus methotrexate group and 9 in the placebo plus methotrexate group, was reported as a retrospective interventional case series [41]. After discontinuation of the study drug at 18 months or earlier, 26 of these 28 participants experienced a relapse of uveitis, after a median time of 150 days. Twenty-five of them initiated adalimumab after their last dose of study drug, including 23 for a uveitis relapse. Twelve participants randomized to adalimumab completed the SYCAMORE trial, and 11 of these 12 had a recurrence by a median of 188 days of extended follow-up. In the placebo arm, only one participant completed the SYCAMORE trial, and 7 of the 9 participants in this group were started on adalimumab for relapsed uveitis.

#### NCT01385826 (ADJUVITE)

The efficacy of adalimumab in patients with refractory active idiopathic anterior uveitis or JIA-associated uveitis was assessed in ADJUVITE, a placebo-controlled study that employed laser flare photometry to measure control of the inflammatory activity in the anterior segment [42]. The primary endpoint was reduction of anterior chamber cells by at least 30% at 2 months, without worsening of anterior chamber cell count or protein flare on clinical examination. The study had a subsequent 10-month open-label phase, during which all patients received adalimumab. Of the 32 patients enrolled, 56% of the adalimumab group responded to treatment, comparing favorably with 20% of the placebo group. According to the Standardization of Uveitis Nomenclature criteria of anterior chamber cell and protein flare grading, there were no significant differences observed between groups at 2 months. No serious adverse reactions related to adalimumab were reported at one year. The authors noted that improvement on adalimumab was faster when the uveitis was more severe, suggesting the early assessment of effectiveness may have masked a potentially higher response rate. However, given the small sample size, they also recommended some caution in interpretation of the positive results of this study.

### Gevokizumab

#### NCT01965145 (EYEGUARD B)

Gevokizumab, a humanized anti-interleukin (IL)-1beta monoclonal antibody, was examined against placebo when added to conventional immunomodulatory drugs and oral prednisone for the treatment of Behçet uveitis [43]. The study enrolled 80 patients but ended after a pre-specified number of ocular exacerbations had occurred, as gevokizumab did not significantly extend the time to first ocular exacerbation (equating to 35% uveitis flares in both treatment groups during the event-driven core study period). Patients in the gevokizumab-treatment arm had a statistically significant reduction in loss of best-corrected visual acuity versus the placebo group (10 ETDRS letters or more: 10% versus 27%). Trends favored gevokizumab in reducing the annual incidence of first ocular exacerbation, and in lowering the incidence of macular edema, retinal infiltrates, and retinal vasculitis. Systemic adverse reactions were similar between the groups, mainly upper respiratory infections, with no re-activation of tuberculosis reported during follow-up.

### Secukinumab

#### NCT00995709 (SHIELD), NCT01095250 (INSURE) and NCT1032915 (ENDURE)

Three randomized placebo-controlled clinical trials–SHIELD, INSURE, and ENDURE – evaluated secukinumab, a fully human anti-IL-17A monoclonal antibody, as an adjuvant therapy for uveitis [44]. The SHIELD study included patients with resistant intermediate, posterior and pan- uveitis associated with Behçet disease; INSURE involved those with active non-infectious uveitis without Behçet disease; ENDURE was directed toward quiescent non-infectious uveitis without Behçet disease. The SHIELD study did not meet its primary endpoint of reducing the recurrence rate of uveitis over 24 weeks. However, secukinumab-treated patients showed a significant reduction in the use of concurrent immunomodulatory drugs. About 18% of patients discontinued the trial, mostly due to adverse events. Confounding factors included the high number of baseline immunomodulatory drugs and the severity of underlying conditions, with frequent exacerbations of uveitis. Due to the negative results in the SHIELD trial, the INSURE trial was terminated early. Similarly, interim data from the ENDURE trial failed to meet the primary endpoint of time to first uveitis recurrence, leading to early termination.

## Phase III clinical trials comparing therapeutics delivered locally to the eye and systemic immunomodulatory drugs for non-infectious uveitis

Two phase III clinical trials compared the surgically placed intravitreal fluocinolone acetonide implant against systemic immunomodulatory drug therapy in patients with non-infectious uveitis involving the posterior segment: the MUST Trial [45–47] and NCT00468871 [48]. In both clinical trials, the two treatment approaches reduced intraocular inflammation. Nearly all phakic patients who received the implant required cataract surgery, and surgery to control elevated intraocular pressure was also common in this group. Systemic immunomodulatory drug therapy was generally well-tolerated and safe.

### NCT00132691 (MUST)

The MUST Trial was planned as a 24-month study of 255 participants (479 eyes) with severe active or recently active non-infectious intermediate, posterior, or pan- uveitis, who were randomized to treatment with an intravitreal fluocinolone acetonide implant (0.59 mg) or systemic immunomodulatory drug therapy (oral prednisone and conventional immunomodulatory drugs, as required) [45]. The MUST Follow-up Study extended an observation period out to between 7 and 10 years, using similar treatment approaches but no mandated protocol. Outcomes were reported at 2 [45], 4.5 [46], and 7 [47] years, with nearly 90% retention at 24 months and around 70% retention at 7 years, despite 20% crossover on treatment.

The primary outcome of change in best-corrected visual acuity at 24 months was not significantly different between the treatment groups: approximately 6 versus 3 logMAR letter gain for eyes in the fluocinolone acetonide implant versus systemic therapy, respectively [45]. By 7 years, there was a modest benefit for the systemic therapy group over the implant group (7 letters), with the caveats of a 30% follow-up loss and non-standardized re-implantations [47]. Only 25% of the originally implanted eyes received an implant in the final 3 years. Both treatments controlled inflammation effectively, typically within 9 months, although the fluocinolone acetonide implant group had significantly lower uveitis activity at 24 months (12% versus 29%). At 24 months, this group also had a greater reduction in central macular thickness (180 μm versus 109 μm). At 24 months, 86% of patients in the systemic therapy group required immunomodulatory drugs to maintain the prednisone at or below 7.5 mg/day. Re-treatment was generally needed after 5 years in the implant group. By 7 years, 18% of eyes in the systemic therapy group also had received an implant.

Local adverse events were more common for eyes in the fluocinolone acetonide implant-treated group than the systemic immunomodulatory drug therapy group, with a significantly higher risk of glaucoma (17% versus 4%) and a high rate of incisional surgeries to manage the intraocular pressure (45% by 7 years) [45, 47]. Over 90% of phakic eyes in the implant group developed cataracts within 24 months, but post-surgery outcomes were similar between groups, and generally good [49]. In the systemic therapy group, 43% required cataract surgery over 7 years, with a prednisone dose over 7.5 mg/day significantly increasing the likelihood of surgery [50]. Both 24-month and 7-year analyses affirmed the safety of systemic therapy, showing no significant differences in hypertension, hyperlipidemia, or fracture rates compared to the implant group, although infections requiring antibiotics were more frequent in the systemic therapy group. Both groups experienced an improved quality of life over 36 months, with implant-treated participants having faster gains [51]. Visual impairment negatively affected quality of life, and a gain of 5 ETDRS letters of best-corrected visual acuity was associated with significant improvement. Systemic immunomodulatory drug therapy was more cost-effective over 36 months; however, for unilateral disease, the fluocinolone acetonide implant was reasonably cost-effective in comparison to systemic therapy [52].

### NCT00468871

The safety and efficacy of an intravitreal fluocinolone acetonide implant (0.59 mg) was also compared against oral prednisolone and systemic immunosuppressive drugs in a 36-month randomized controlled trial of 140 patients with non-infectious posterior uveitis [48]. Patients with both unilateral and bilateral disease were included; for bilateral disease assigned to the implant, the more severely affected eye was implanted, and the less affected eye received periocular injections. This differed from the MUST Trial, in which both eyes received the assigned intervention [45]. The study had a strong retention rate, with 94% completing the 24-month visit. At 24 months, mean visual acuity was similar between groups, with 75% maintaining stable vision and 15% improving by 3 or more ETDRS lines. The implant group showed more fluctuations, with declines in visual acuity around the time of implantation and at 15 to 18 months, coinciding with cataract development in phakic eyes. In comparison to systemic immunosuppressive drug therapy, the implant provided better inflammation control, with fewer recurrences (18% versus 64%), longer time to first recurrence, greater median reduction in number of recurrences, and lower vitreous haze scores. Angiographic macular edema was significantly more likely to improve in implant-treated eyes (87% versus 74%).

Cataract and elevated intraocular pressure were common complications for eyes treated with the fluocinolone acetonide implant [48]. By 24 months, just over 20% of implanted eyes required glaucoma surgery, compared to 3% in the systemic therapy group, with most surgeries occurring between week 30 and month 24 post-implantation. Approximately one-half of implanted eyes had intraocular pressure elevations of 10 mmHg or more, versus 10% in the systemic therapy group. Cataract surgery was performed in 88% of phakic implanted eyes, compared to 20% in the systemic therapy group. In contrast, about 25% of patients in the systemic immunosuppressive drug arm experienced non-ocular treatment-related adverse events, most commonly hypertension, while no such events occurred in the fluocinolone acetonide implant group.

## Ongoing phase III clinical trials of treatments for non-infectious uveitis

Many of the anti-inflammatory drugs currently in use for non-infectious uveitis have not been formally compared against placebo or other drugs in phase III clinical trials, and there is no shortage of drugs in the pharmaceutical pipeline targeted to the disease and its complications. Biologic immunomodulatory drugs make up most of the agents reaching clinical trials at the present time. This section briefly surveys ongoing phase III clinical trials of locally delivered therapeutics or systemic immunomodulatory drugs for non-infectious uveitis, with Table 3 providing additional details of these studies.

**Table 3:** Summary of ongoing phase III clinical trials evaluating therapeutics delivered locally to the eye or systemic immunomodulatory drugs for non-infectious uveitis

Identification number (name) [Ref]	Design and location	Intervention	Uveitis subtype	Primary endpoint	Status as of December 16, 2024
NCT02556424 (TRIOZ) [53]	Multicenter, open, randomized, controlled trial France	Subconjunctival triamcinolone injection (16 mg) vs. intravitreal dexamethasone implant (700 µg)	Uveitis with macular edema	Central retinal thickness difference from baseline at 2 months	Completed, without published results
NCT05486468 (TYNI) [55]	Randomized, double-masked, controlled trial United States	Two intravitreal fluocinolone acetonide implants (each 0.18 mg) vs. sham injection	Active PU	Recurrence rate of uveitis at 6 months	Unknown
NCT05642325 (Sandcat) [56] and NCT05642312 (Meerkat) [57]	Multicenter, randomized, double-masked, placebo-controlled trial Sandcat: Argentina, Australia, China, Czech Republic, France, Germany, India, Italy, Japan, Kazakhstan, Portugal, Singapore, South Korea, Spain, Switzerland, Taiwan, Turkey, United Kingdom, United States Meerkat: Austria, Brazil, Canada, China, Israel, Italy, Mexico, South Korea, Netherlands, Poland, Portugal, Taiwan, United Kingdom, United States	Vamikibart (0.25 mg or 1.0 mg) vs. sham	Uveitis with macular edema	Percentage of participants with improvement of ≥ 15 letter in BCVA at week 16	Active, not recruiting
NCT06258915 (FOCUS) [58]	Multicenter, randomized, controlled trial France	Adalimumab vs. mycophenolate mofetil	Active IU, PU, PanU	Rate of treatment failure at 36 weeks	Not yet recruiting
NCT04798755 (Co-THEIA) [59]	Multicenter, randomized, single-masked, controlled trial Spain	Methotrexate + adalimumab vs. methotrexate vs. adalimumab	Active IU, PU, PanU	Complete and maintained resolution of uveitis	Recruiting
NCT06431373 (CLARITY) [62]	Randomized, double-masked, placebo-controlled trial United States	Brepocitinib vs. placebo	Active IU, PU, PanU	Rate of treatment failure on or after 6 weeks up to 24 weeks	Recruiting
NCT05651880 (JAKUVEITE) [63]	Open-label, single group assignment trial France	Baricitinib	Active IU, PU, PanU	Number of patients in complete remission at 6 months	Not yet recruiting
NCT04088409 [64]	Multicenter, open-label, controlled trial France, Germany, Italy, Spain, United Kingdom	Baricitinib vs. adalimumab	JIA-associated uveitis, ANA-positive chronic anterior uveitis	Percentage of responders at 24 weeks	Active, not recruiting

**Abbreviations**: ANA: antinuclear antibody; BCVA: best-corrected visual acuity; IU: intermediate uveitis; JIA: juvenile idiopathic arthritis; PanU: panuveitis; PU: posterior uveitis

### Therapeutics delivered locally to the eye

The TRIOZ randomized clinical trial (NCT02556424) has enrolled up to 142 patients in a protocol that will compare the efficacy and safety of an inexpensive subconjunctival triamcinolone acetonide injection (0.4 mL of 40 mg/mL) versus the intravitreal dexamethasone implant (0.7 mg) for the treatment of uveitic macular edema [53]. The planned primary endpoint is the central retinal thickness difference between baseline and 2 months post-treatment. Relative effectiveness of the subconjunctival triamcinolone acetonide injection would have major economic implications, but the results are yet to be published.

A single intravitreal fluocinolone acetonide implant (0.18 mg) was effective in reducing recurrences, decreasing the need for adjunctive treatments, and limiting visual loss in patients with non-infectious uveitis involving the posterior segment [54]. On that basis, there is an ongoing randomized placebo-controlled clinical trial (TYNI study: NCT05486468) to evaluate the efficacy of two consecutive implants for treating chronic non-infectious intermediate, posterior, and pan- uveitis [55]. The primary endpoint is the recurrence rate of uveitis at 6 months, and at 12 months as a secondary endpoint.

Intravitreal injection of an anti-IL-6 monoclonal antibody, vamikibart, will have safety and efficacy assessed through two large randomized sham-controlled clinical trials (Sandcat and Meerkat studies: NCT05642325 and NCT05642312, respectively) with identical protocols, involving patients with active or inactive non-infectious uveitis and uveitic macular edema [56, 57]. The drug will be injected every 4 weeks until week 12, and PRN from weeks 20 to 40. The primary outcome measure will be the percentage of participants with an improvement in best-corrected visual acuity of at least 15 letters after four treatments, as judged at week 16.

### Systemic immunomodulatory drugs

In an effort to establish the relative roles of conventional and biologic immunomodulatory drugs for treatment of non-infectious uveitis, the FOCUS randomized clinical trial (NCT06258915) will compare mycophenolate mofetil to adalimumab in up to 120 patients with active non-infectious intermediate, posterior, or pan- uveitis [58]. The primary outcome measure will be the rate of treatment failure at 36 weeks. An independent 3-arm clinical trial, Co-THEIA (NCT04798755), will enroll up to 192 patients with active non-infectious uveitis and randomize them to treatment with methotrexate, adalimumab, or the combination of both drugs [59]. Outcomes will be determined based on resolution of inflammation, achieved within the first 16 weeks and maintained until week 52, plus the absence of safety and tolerability treatment failure.

A successful phase II study of the janus kinase family inhibitor, brepocitinib, for non-infectious uveitis [60, 61], has paved the way for the CLARITY phase III randomized controlled trial (NCT06431373) [62]. The new study will enroll an estimated 300 patients with non-infectious uveitis that is active in the posterior segment of the eye to establish time to treatment failure for brepocitinib versus placebo.

Two small phase III clinical trials will evaluate another janus kinase family inhibitor, baricitinib, for different forms of non-infectious uveitis. The JAKUVEITE trial (NCT05651880) will evaluate the efficacy of baricitinib with patients with non-infectious non-anterior uveitis refractory to one or more other biologic immunomodulatory drugs [63]. Patients must stop their concurrent immunosuppressants at least 10 days before the start of treatment, and the primary outcome measure is the number of patients with uveitis in complete remission at 6 months. A second trial (NCT04088409) will compare baricitinib with adalimumab in children and adolescents with JIA-associated uveitis or JIA-type anterior uveitis without systemic manifestations, and an insufficient response to methotrexate [64]. The primary endpoint measure is the percentage of responders at week 24 after start of treatment.

## Conclusions

Non-infectious uveitis is an uncommon inflammatory eye disease, but the cause of substantial vision loss and blindness globally. This review has described a range of drugs and therapeutics that have been evaluated for effective and safe treatment of non-infectious uveitis in phase III clinical trials. The majority of therapeutics delivered by local injection to the eye, or surgically positioned inside it, are corticosteroid-based. Trials have shown these to be effective for reducing intraocular inflammation, with complications that include elevated intraocular pressure and glaucoma, as well as cataract. Although a wide range of systemic immunomodulatory drugs are used to treat non-infectious uveitis in clinical practice, well under one-half have been studied in phase III clinical trials. These trials have provided key pieces of information including the following: similar effectiveness of methotrexate and mycophenolate mofetil for intermediate, posterior, and pan- uveitis; usefulness of interferon-beta for intermediate uveitis; and potent anti-inflammatory activity of adalimumab in recalcitrant uveitis. Accurate dosing and careful monitoring are essential aspects of using systemic drug treatments. They have been defined largely by medical specialties outside ophthalmology, and are presented in Table 4. The ‘Fundamentals of Care for Uveitis’ initiative provided consensus guidelines for implementation in the management of non-infectious uveitis specifically [65].

**Table 4:** Description of systemic immunomodulatory drugs evaluated for non-infectious uveitis in phase III clinical trials

Drug	Drug class	Mechanism of action	Screening investigations	Dose	Adverse effects	Notes
Methotrexate	csDMARD: anti-metabolite	Inhibition of enzymatic pathways including aminoimidazole, caroboxamide, ribonucleotide, transformylase [84]	CBE, LFT, hepatitis serology, HIV serology, IGRA +/- liver ultrasound, CXR*	10–25 mg, PO, once weekly	**Clinical**: Gastrointestinal symptoms **Laboratory**: Cytopenia, elevated liver enzymes	Assess CBE, LFT every 2–4 weeks initially, then 6–8 weekly thereafter [85] Risk of infection, including reactivation of pulmonary tuberculosis [86] Contraindication in pregnancy due to risk of birth defects [86]
Mycophenolate mofetil	csDMARD: anti-metabolite	Inosine monophosphate dehydrogenase inhibitor [84]	CBE, LFT, hepatitis serology, HIV serology, IGRA	1000–3000 mg, PO, divided dosing, daily	**Clinical**: Gastrointestinal symptoms **Laboratory**: Cytopenia	Assess CBE, LFT every 2–4 weeks initially, then 6–8 weekly thereafter [87] Contraindication in pregnancy due to risk of birth defects [88]
PEG-Interferon alpha-2a (form of interferon currently available)	bDMARD: cytokine	Pleotropic effects including blocking leukocyte migration, transcriptional modulation of chemokines and cytokines, direct action on T cells [89]	CBE, LFT, renal function	Initial dose 180 mcg, SC, once a week, titrated down on response	**Clinical**: Coryzal symptoms, cutaneous manifestations including erythema **Laboratory**: Leukopenia, neutropenia, elevated liver enzymes	Assess CBE, LFT and renal function every 2–4 weeks initially, then 6–8 weekly thereafter [90] Periodic assessment of thyroid function tests due to risk of thyroid dysfunction [91]
Adalimumab	bDMARD: anti-cytokine monoclonal antibody	Prevents action of TNF-alpha, through binding both transmembrane and soluble forms, Fc-mediated effector function such antibody dependent cellular cytotoxicity and complement mediated cytotoxicity [92]	CBE, LFT, hepatitis serology, HIV serology, IGRA, CXR	Initial dose 80 mg, SC, followed by 40 mg, every 2 weeks	**Clinical**: Injection site reactions **Laboratory**: Cytopenia, elevated liver enzymes	Assess CBE and LFT every 6–8 weeks [93] Risk of infection, including respiratory tract infections and reactivation of pulmonary tuberculosis [34] Adalimumab levels for adherence [94] Anti-adalimumab antibody levels if drug loses efficacy [94]
Gevokizumab	bDMARD: anti-cytokine monoclonal antibody	Binds to IL-1beta, preventing its interaction with receptor, IL1R1 in which activation of intracellular inflammatory signaling pathways are inhibited [95]	CBE, LFT, hepatitis serology, HIV serology, IGRA, CXR	60 mg, SC, every 4 weeks	**Clinical**: Headache, myalgia [96] **Laboratory**: Nil identified	Risk of infection, including respiratory tract infections [95]
Secukinumab	bDMARD: anti-cytokine monoclonal antibody	Binds to IL-17A, preventing its interaction with its receptor, IL-17R leading to inhibition of pro-inflammatory cytokine release [97]	CBE, LFT, renal function, IGRA, CXR, hepatitis serology, HIV serology, ANA, dsDNA, pregnancy test	30 mg/kg, IV, or 300 mg, SC, every 2 weeks	**Clinical**: Gastrointestinal symptoms, cough and rhinorrhea, urticaria [98, 99] **Laboratory**: Neutropenia	Risk of infection, particularly candidal infections of upper and lower respiratory tract as well as reactivation of pulmonary tuberculosis [99] Assess CBE, serum electrolytes, renal function, LFT, urinalysis at 3 months from initiation of treatment, then every 6-months [100]
Brepocitinib	tsDMARD	Selective JAK1 and TYK2 inhibitor, resulting in inhibition of pro-inflammatory cytokines, particularly IL-17 [101]	CBE, LFT, renal function, IGRA, CXR, hepatitis serology, HIV serology	30–60 mg, PO, once daily	**Clinical**: Acne, gastrointestinal symptoms [102] **Laboratory**: Elevated liver enzymes and creatinine, cytopenias – lymphopenia, neutropenia [101, 102]	Risk of infection, particularly respiratory tract [103]

**Abbreviations**: ANA: antinuclear antibody; bDMARD: biologic disease modifying anti-rheumatic drug; CBE: complete blood examination; csDMARD: conventional synthetic disease modifying anti-rheumatic drug; CXR: chest x-ray; dsDNA: double stranded deoxyribonucleic acid antibody; HIV: human immunodeficiency virus; IGRA: interferon gamma release assay; IL: interleukin; IV: intravenously; JAK: janus kinase; LFT: liver function test; PO: orally; SC: subcutaneously; TNF: tumor necrosis factor; tsDMARD: targeted synthetic disease modifying anti-rheumatic drug

*Liver ultrasound recommended for patients with metabolic syndrome or elevation in liver enzymes suggestive of fatty liver disease. This is a relative contraindication to methotrexate administration, due to risk of worsening of existing disease. CXR considered in those patients with moderate-high risk of tuberculosis exposure

In the field of uveitis, phase III clinical trials are limited in number in comparison to their volume in other areas of ophthalmology and medicine. For the most part, however, findings of published trials on uveitis treatment are rapidly implemented into general clinical practice. For example, the report by the ‘International Study Group for Systemic Immunomodulatory Drug Treatment of Non-Infectious Uveitis’ demonstrated that methotrexate and adalimumab were the most common first-line conventional and biologic systemic drug choices, respectively [6], consistent with positive effectiveness and safety evaluations in randomized clinical trials [30, 34, 36]. Real-world studies for locally delivered therapeutics similarly indicate widespread use of the short-acting dexamethasone and long-acting fluocinolone acetonide implants [18, 66, 67], as supported by randomized clinical trials [7, 11]. Clearly the availability of, and access to, different anti-inflammatory drugs and therapeutics varies around the world, and this naturally will impact translation of clinical trial findings into practice. From this perspective, it is encouraging to note that the latest World Health Organization Model List of Essential Medicines includes both methotrexate and adalimumab [68]. The intravitreal dexamethasone implant is not on this list, but it is available in many countries around the globe [69], and intravitreal injection of triamcinolone acetonide remains an inexpensive, widely available alternative [8].

Both new drug targets for non-infectious uveitis and novel routes of drug delivery should be anticipated in the foreseeable future. Over the past several decades there have been major advances in knowledge of the specialized immune-privileged ocular microenvironment, and in understanding of basic mechanisms of intraocular inflammation and its complications [70, 71]. This understanding is already translating to new biologic drugs that target disease processes. New drugs may be adopted from their use for non-eye inflammatory diseases or developed for uveitis *de novo*. The potential targets include a plethora of inflammatory cytokines, cell adhesion molecules, eicosanoids, and intracellular signaling proteins [72–75], as well as other molecular mediators. Beyond the molecule itself, therapeutic options include targeting the receptor or the encoding transcript. For example, we have shown that transcription of the endothelial adhesion molecule, intercellular adhesion molecule 1, can be targeted to limit leukocyte interactions with the vascular endothelium, an initiating stage in non-infectious uveitis [76]. Developments in the area of local delivery for drugs are numerous, and include eye drops with high intraocular penetration, new intraocular implants that have reservoirs for re-fill, plus topical treatments combined with iontophoresis [77–79]. Gene therapy has already reached the clinic for correcting mutations in inherited retinal diseases, and now is being considered as a means to deliver anti-inflammatory agents to the eye in non-infectious uveitis [80].

Ophthalmology is one of the most visual of all medical specialties, and another area of advancement that is likely to impact future phase III clinical trials is multimodal imaging of the eye. Today, the spectrum of ocular imaging technologies includes fluorescein angiography, indocyanine green angiography, different optical coherence tomography modalities with and without angiography, fundus autofluorescence, and near infrared imaging, as well as color fundus photography. Ocular imaging provides the opportunity to further the accurate measurement of intraocular inflammation, and almost certainly will be increasingly incorporated into outcome measures of new phase III clinical trials. Already some studies are using central retinal thickness measured by optical coherence tomography as the primary outcome measure [28, 29]. The ‘Multimodal Imaging in Uveitis’ project was recently initiated to develop clinical classifications and diagnostic criteria for use in clinical trials, as well as for daily practice [81].

Emerging drugs and therapeutics hold substantial promise for improving visual outcomes and quality of life in patients with non-infectious uveitis. Future clinical trials will be instrumental in defining the efficacy and safety of these novel treatments, paving the way for their integration into clinical care protocols. Projecting 5 years out from the present day, we expect to see interest in defining treatments for specific subsets of uveitis, following on from outputs of the Standardization of Uveitis Nomenclature project that provide classification criteria for 25 uveitides [3]. As non-infectious uveitis is an uncommon disease, phase III clinical trials for this condition face challenges in enrolment and thus are frequently international by necessity. Almost certainly new trials will draw on artificial intelligence, particularly in areas such as development of clinical trial eligibility criteria, enhancement of participant diversity, and reduction in sample sizes [82]. In addition, we hope to see the use of core outcome measures for productive cross-referencing of results of all phase III clinical trials for non-infectious uveitis [83].

## Data Availability

No datasets were generated or analysed during the current study.

## Abbreviations

ETDRS: Early Treatment Diabetic Retinopathy Study
FDA: United States Food and Drug Administration
IL: interleukin
JIA: juvenile idiopathic arthritis
logMAR: logarithm of the minimum angle of resolution
PRN: pro re nata
