# Publisher Correction: Pharmacotherapy for non-infectious uveitis: spotlight on phase III clinical trials of locally injected or implanted therapeutics and systemic immunomodulatory drugs

**DOI:** 10.1186/s12348-025-00513-6

**Published:** 2025-07-17

**Authors:** Melissa K. Shields, Lisia Barros Ferreira, Syed B. Ali, Liana Dedina, Lyndell L. Lim, Eric B. Suhler, Justine R. Smith

**Affiliations:** 1 College of Medicine and Public Health, and Flinders Health and Medical Research Institute, Flinders University, Adelaide, South Australia Australia; 2 Centro de Medicina Humanizado, Curitiba, Paraná Brazil; 3 Flinders Medical Centre, Southern Adelaide Local Health Network, Adelaide, South Australia Australia; 4 Centre for Eye Research Australia, Melbourne, Victoria Australia; 5 Casey Eye Institute, Oregon Health & Science University, and Portland Veterans’ Affairs Health Care System, Portland, OR USA

**Publisher Correction** **:** **Journal of Ophthalmic Inflammation and Infection 15, 49 (2025)**

**https://doi.org/10.1186/s12348-025-00502-9**

Following publication of the original article, it came to the journal's attention that the contents of the Competing Interests declaration had been duplicated. This duplication has now been corrected. The publisher thanks for you reading this erratum and apologizes for any inconvenience caused.

